# Supplementary material for: Immiscible inclusion bodies formed by polyglutamine and poly(glycine-alanine) are enriched with distinct proteomes but converge in proteins that are risk factors for disease and involved in protein degradation
Source: PLoS One. 2020 Aug 28;15(8):e0233247. doi: 10.1371/journal.pone.0233247 (PMC7455042; doi:10.1371/journal.pone.0233247)
Supplement: Attachment — Submitted filename: response to reviewers.pdf [file pone.0233247.s008.pdf]

Dear Prof van der Wel,

We thank the reviewers for their feedback and comments. We have made major changes to the manuscript to address both the reviewers queries and also to improve the readability of the manuscript. These changes are outlined below.

Please provide a point-by-point response to all the comments identified by the authors. One key question relates to the comments from reviewer 3, who notes that the different experiments were performed at distinct time points, which may affect the ability to integrate the respective findings. The reviewers also comment on a need to clarify or rephrase a number of claims made, including in the abstract. In particular, there are some questions about whether the reasoning or evidence behind certain mechanistic claims are completely clear. Among other significant questions, I also note some issues where the text appears to be unclear about which conditions or samples are being compared in comparative statements in the paper (see reviewer comments).

1. Please see our responses below that address these critical questions.

Reviewer #1: In the article titled, "Inclusion bodies formed by polyglutamine and poly(glycine-alanine) are enriched with distinct proteomes but converge in proteins that are risk factors for disease and involved in protein degradation", Radwan and coworkers use state-of-the-art proteomics techniques to evaluate the proteins that co-purify with specific proteinaceous aggregates. They focus on a polyglutamine-expanded fragment of Huntingtin protein exon 1 (Httex1Q97) and a glycine-alanine expansion (polyGA101), which respectively are models for Huntington's disease and ALS (caused by expansions in C9orf72). These pathological proteins were expressed in cell culture and the authors isolated the aggregates for proteomic and bioinformatic analyses. The major observation is that these pathological proteins formed distinct aggregates within cells and each type of aggregate contained a unique set of enriched co-aggregating proteins. Thus, the aggregation of each species has the potential to interfere with cell machinery differently and cellular pathology may occur via protein-specific mechanisms. The authors also include an important control for polyGA101 in which they determine that co-aggregation of specific proteins is not a result of protein over-expression.

Overall, the work is clearly presented and provides new data sets for understanding potential pathological mechanisms associated with protein aggregation. The authors provide some speculation about the significance of certain co-aggregating proteins, but this analysis seems incremental in its advancement of knowledge. However, the authors do not over-interpret their data.

Questions for authors:
Were there any proteins that were identified only in HttexQ97 or polyGA101 samples? The Supplementary Table 2 suggests the authors identified 737 total proteins and all of these proteins were present in both samples. Perhaps the enriched proteins that were discussed in the text only make up a small percentage of the proteins that were co-aggregating in both types of samples? If that were the case, their significance would be diminished. It also seems possible that some proteins could be completely specific to only one type of aggregate? Making these points clearer in the text might make it easier for the reader to interpret.

1. In the original manuscript we noted a lack of proteins specific to each inclusion class. However, we realised that we had inadvertently excluded potential candidates from analysis

because proteins that are missing from one of the inclusions (but are present in the other) would not have passed our statistical filtering steps since they are reported as missing (blank) values by the proteomics software. To address this issue we applied a standard proteomics analytical process called zero-filling, which involved the reinspection of the missing values of all proteins and replacing them with a small constant value to enable these values to be considered in the statistical analysis. This resulted in 7 proteins being found to be unique to polyGA inclusions and 4 proteins unique to Httex1Q_{97} and many other proteins with changed enrichment ratios. We have modified the abstract, methods and results sections to explain the methods, results and conclusions of the revised analysis. This includes updating the data presented in Fig 2 and Table S2 to highlight these changes and show the unique proteins.

Major suggestion:
A more quantitative presentation of the data in Figure 1B is needed. It is not clear how representative this image is of its larger cell population.

2. We have included an additional lower magnification image in Fig 1B to give a clearer indication of how representative the data is.

Minor suggestions:
On Line 243, the authors should emphasize that the proteins are enriched relative to the other type of aggregate (Htt vs polyGA). It's possible to read this paragraph and think the proteins are enriched over "background".

3. We have made this modification.

In the Figure 1A legend, specify the number of cells used for the graph and include the p-value.

4. Please see response 16 below. As a result of those changes we have now deleted Fig 1A and hence this query is now redundant.

On Line 276, I think the authors intend to reference Figure 2C.

5. We thank the reviewer for identifying this error. We have now changed this to Fig 2B, which is what the reviewer meant.

Reviewer #2: This manuscript by Radwan and colleagues describes a well-designed set of experiments to determine the differences in the composition of polyQ and polyGA inclusions and to study the differences in the total proteome of cells with and without polyGA inclusions. It will provide a valuable resource for researches in the fields of both Huntington's Disease and Frontotemporal dementia/amyotrophic lateral sclerosis. I recommend this manuscript for publication without further experiments, but I believe that a few points need to be explained/described in more detail:

It is not clear to me why the authors describe the disease caused by C9ORF72 GGGGCC hexanucleotide repeat expansion as motor neuron disease and not as Frontotemporal dementia and/or amyotrophic lateral sclerosis 1. This decision should be explained in the manuscript, and the connection between C9orf72 and FTD/ALS has to be mentioned prominently.

6. We agree that this is a more specific description of the disease and we have modified the manuscript to change to this nomenclature of disease.

The abstract mentions that “both structures revealed a synergy of degradation machinery” and in the last paragraph it is stated that the “mechanisms of protein clearance mechanism appear relevant to both aggregation types”. It is not clear to me what is meant by that, and I have a hard time finding data addressing this point in the results section of the manuscript. It is shown that the proteasome is enriched in polyGA aggregates, but I can’t find any other degradation pathways regarding polyQ.

7. We thank the reviewer for highlighting this important point which we agree was not as clear as it needed to be. To make these points clearer we have added a new panel to Fig 2 to show 11 key significant Gene Ontology terms. 7 of these terms contained enriched proteins for both Httex1 and polyGA that centre on stress, protein folding and degradation (cytoplasmic stress granule, ubiquitin-dependent protein catabolic process, unfolded protein binding, ERAD pathway, P-body, and ubiquitin-dependent ERAD pathway). We have also modified the text to explain these details more clearly.

Previous studies on the composition of polyQ and polyGA inclusion bodies should be mentioned more prominently, including the work from N. Nukina that also used FACS sorting to isolate polyQ inclusions (Mitsui et al 2002).
While not absolutely necessary, I think it would increase the value of this study if the overlap and differences between the published datasets and these new datasets would be discussed.

8. Misui et al identified 8 prominent proteins in purified Httex1 inclusions that eluted by SDS-PAGE (HSP84, HSC70, α-tubulin, β-tubulin, EF-1α, HDJ-1 and HDJ-2 and actin). We observed all of these in our study. HDJ-1 and HDJ-2 are Hsp40 family protein members and we observed significant enrichment of Hsp40 proteins dnaja2, dnajb1 and dnajc9 in the polyQ inclusions (Table S2). Of the others identified by Misui et al we saw no specific enrichment to polyQ inclusions, which suggests that these proteins are recruited to both inclusion types. This conclusion is supported by the enrichment of HSP84 (which is Hsp90a) and EF1α (which is Eef1a) immunoreactivity to the surface of the inclusions in the Mitsui et al study. We have added this discussion to our manuscript.

The sentence in 274 needs clarification. “Overall both inclusions yielded an enrichment for gene ontology and KEGG networks of microtubule cytoskeleton, proteasome complex, chaperones, RNA splicing and nuclear envelope (Fig 2C; Table S3).” To me this sounds like both inclusions are enriched in these pathways whereas Figure 2 and Table 2C show GO terms enriched in either one kind of inclusion or the other.

9. We thank the author for pointing out this ambiguity and we have revised the text to clarify our meaning.

GFP is among the enriched proteins in the polyGA interactome, and potentially 2 times more polyGA could also mean two times more background binders. The authors should explain in more detail how this can influence their analysis, and what steps have been taken to control for that. I don’t think that it is invalidating the analysis, but I believe it is important to discuss potential problems.

10. We have now added more discussion to address this point. The enrichment of GFP to the polyGA aggregates suggests that the inclusions formed by polyGA contain less associated proteins than that for Httex1. This could arise by more non-specific interactions to the Httex1 inclusions (as suggested by the reviewer) or by polyGA forming a greater proportion

(by mass) of the inclusion composition than for Httex1 inclusions. Both points are now discussed in the manuscript.

The authors argue that the Go terms listed in 3C and S5 indicate a stress response. Can they explain what they mean with this, and give some references where these terms are connected to stress response pathways.

11. We have now expanded this section to include a more complete description and additional references. In essence, some of these pathways would be anticipated to be correlated to stress responses incurred by protein aggregation or proteostasis imbalance based on links between ribosome biogenesis and nucleolar stress response (Pfister, 2019), ribosome biogenesis and proteostasis imbalance (Tye, 2019), mitochondria as mediators of apoptosis (Wang, 2009), and role of the Golgi as a stress sensor in neurodegeneration (Machamer, 2015).

Minor points:
- Sentence in line 41/42 is incomplete

12. We have revised the sentence to correct the grammatical error.

- 276: The Figure described is not 2C.

13. We thank the reviewer for identifying this typo, which is now fixed.

- Sentence in line 345/346 is incomplete

14. We have revised the sentence to correct the grammatical error.

Reviewer #3: In this current article, the authors evaluate proteinaceous inclusions known to be present in C9orf72-ALS and Huntington's Diseases. Initially, they investigate the time of formation of C9orf72-ALS associated poly-GA inclusions and show that their rate of formation is slower than that of poly-Q aggregates found in Huntington's. Following isolation of these aggregates, the authors then showed an impressive mass spectrometry analysis of proteins enriched in these two instances, along with STRING interaction maps showing involved cellular pathways. The authors also then provided a secondary mass spectrometry analysis, using the novel PulSA method of distinguishing between cells containing diffuse and aggregated poly-GA. The results from these various experiments will be valuable in identifying protein targets and pathways involved both in Huntington's, C9orf72-ALS, and potentially a broader range of neurodegenerative diseases involving protein degradation.

Strengths:
The abstract and introduction are both concise and informative, detailing all key findings of the paper. The manuscript details appropriate inclusion of statistical methods, availability of data sets to the public, and justification of replicates and controls used. Figure 1 demonstrates an impressive ability to image and evaluate the same cells every 15 minutes for 4 days straight. Figures 2A and 3B both show distinct proteomal enrichments with striking volcano plots. Key proteins and pathways are identified associated within poly-GA and polyQ inclusions, which will be informative in guiding future studies and potential therapeutics for these diseases.

Major points of concern:
The use of PulSA is novel and allows for meaningful and insightful results in the given manuscript. However, it is unclear why there is a shift in timing from the 24hr collection of aggregates for "aggregate enriched" mass spec (starting line 91), and a later collection time of 48hr for PulSA analysis (starting line 107). There is no justification for this in the text. The highlighted takeaway from Figure 1 was that very few GA expressing cells has detectable GA aggregates at the 24hour timepoint, and that almost all had formed inclusions by 60hr. It is confusing therefore as to why the 24 timepoint was used for the staining of inclusions in Figure 1 part B, as well as the aggregates purifies for mass spec in Figure 2 when so few inclusions were present. The timepoint then shifted to a 48-hr timepoint for the GA-influence proteome mass spec evaluated in Figure 3, without a rationale or explanation.

15. First, the differences in the timings are the result of these experiments being performed in context of a much wider set of studies that were carved off to a different publication (Radwan et al (2020) Mol Cell Proteomics 19, 640-654). Hence, they are a legacy experimental design, but we do make it clear in the manuscript as to how it was done (as indicated by the reviewer). Second and more importantly, with respect to the data in Figure 1, we realized that we had not explained the experiment clearly that could lead to confusion as to what the data means and implications to how much aggregation has occurred. To clarify the time course of aggregation in Fig 1A (of the original submission) only counted inclusions that formed *after* the 24 hour time point. We did this because it was not possible in the design of the experiment to fully measure the rate of aggregation at earlier timepoints. However, by 24 hours a significant proportion of the cells had formed aggregation (estimated at 20 %). Note that in the figure we had set the 24 hour time point to zero inclusions because we only counted cells from that time point on without inclusions. It is important to note that because of this the data in Fig 1A is really not comparable to any of the other experiments that were performed in the manuscript, which we now appreciate in hindsight is problematic. We have therefore decided to remove the time course data (Fig 1A in the original submission). Removing this data does not change any important conclusions of the manuscript.

More explicit description is required regarding the "whole proteome" alterations associated with GA aggregation for Figure 3B. On the basis of the in-text and figure legend description, it seems that the comparison that was performed is solely the proteome changes that occur between cells containing GA soluble versus GA insoluble inclusions. However, the volcano plot in Fig 3B seems to also compare the proteome outcomes of these two GA states to GFP alone. It is pivotal to understand which of these possibilities has been evaluated. Proteomic changes are likely to occur comparing GA soluble to GFP or control non-transduced cells, and so this must be explicitly addressed. Evaluation and consideration of the publicly available dataset related to Figure 3B needs to be done in light of the actual experimental setting performed.

16. To clarify, the experiment only compares cells containing GA soluble versus GA insoluble inclusions. The GFP label on the figure refers to abundance of the GFP moiety that arises from proteomic digestion from GA-GFP fusions and hence is an estimate of the abundance of the fusion (not GFP as indicated). Because this label may lead to the readers thinking the experiment compares to GFP cells we have changed the labels in all the figures to more clearly specify that this is the case. We have also revised the text in the results section and figure legend to more clearly explain how the experiment was performed.

Minor points of concern:
The introduction was thorough yet brief. Overall it seems that the proteome changes of poly-GA

versus the arginine-containing DPRs should be highlighted a bit more. Furthermore, presenting a stronger case for this study comparing poly-GA versus poly-Q will be meaningful, given the very distinct pathologies of C9-ALS versus Huntington's Disease, and that the overall proteome changes for both individual disease phenotypes have already been established in Neuro2a cells and primary neurons.

17. We thank the reviewer for this suggestion which made us realised we had not clearly articulated the rationale for comparing polyQ with polyGA. Our major justification was to examine whether two unrelated proteins that form superficially similar-appearing structures had similar co-aggregation mechanisms. We have now substantially revised the text to clarify this which includes new sections in the introduction.

It is unclear as to why additional proteins are included to left of the STRING interaction maps in Figure 2B, this should be noted in the figure legend. Presumably this figure includes all 48 polyQ-enriched and all 69 poly-GA enriched proteins from portion A.

18. These proteins are present in the dataset but have no known protein-protein interactions with the other proteins shown. We have now more clearly indicated this in the figure legend.

The prior known association of poly-GA dipeptides with SQSTM1/p62 should be noted either in line 270, or in the description of Figure 2B results (May Acta Neuropathol 2014).

19. We have added this reference to the manuscript.

The paragraph involving Httex1Q97 enriched proteome (starting line 290) could be expanded upon with implications.

20. We have added additional information in this section.

The "in-text" description of the exclusion of cells for Figure 3 is unclear (line 299). It should be clarified within the main text, as was done in the figure legend (line 321), to allow for better understanding of which cells were actually evaluated.

21. We have now expanded this section to more completely explain the details.

Does the statement in lines 300-304 "A lower yield of aggregates than we measured by live cell imaging" imply that some cells recorded in the live imaging experiments were actually "almost dead" and would have been Sytox positive if tested?

22. We have now revised this section of text to clarify the meaning.

The statement "there was no overlap in the proteins seen enriched in polyGA inclusions with proteins that changed expression due to polyGA aggregation" requires an explanation into how this comparison was performed.

23. We have now revised this section of text to clarify the comparison.

The authors mention "lastly we investigated the overlap of proteins enriched in Httex1Q97 inclusions" (line 333). While there is a description of the results and highlighting of key findings, there is no figure representing this data.

24. We have now substantially revised this section to include a new figure (Fig 4) and new analysis of the data to investigate the overlap of the previous data with both proteins enriched in the Httex1 inclusions and proteins identified in our data as candidates for binding both Httex1 and polyGA inclusions. The new analysis shows a greater connection to the broader conclusions linking the data to mechanisms of protein misfolding and clearance.

The description of Picalm found in polyQ inclusions, and the link to Alzheimer's disease is an interesting one (line 345-349). As the implication is a "broad link to neurodegenerative diseases" it would also be intriguing to know if Picalm was also found in the poly-GA inclusion protein list.

25. To clarify Picalm is among proteins found in both polyGA and Httex1 inclusions. The data in Table S3 provides an indication of the relative enrichment towards the polyGA and Httex1 inclusions. Refer also to response 2 above.

Conclusion: The statement starting on line 352, "the mechanisms of protein clearance" needs to be highlighted more specifically with examples. This seems to be one of the key conclusions that the authors wish to put forward, however it is not extremely well supported from the reading of the text at this time. Additionally, at the end of this statement it would be beneficial to include of list of the mutated proteins which are modifiers of MND risk (line 354). Overall implications and next steps also are lacking, as to how this data may be applied to future studies.

26. We believe the changes we have made in response to other questions now better explain the justification for our conclusions. Please refer to responses 8, 12 and 25 above. We have now included the risk of genes as a new Supplementary Table (Table S7).

Overall, the paper would benefit from a thorough proofreading to rectify the following errors and potentially more that the authors will identify:

• Methods: NanESI-LC-MS analysis. Starting at line 156, it is unclear what "B" is. It seems that it may be 100% CH_3CN/5% DMSO/0.1% formic acid v/v as listed. The end of this line also states "B" to "B" multiple times in describing the percentages of solutions used for an elution gradient. Should these say A to B, or are B different percentages of eluent B truly used?

27. We have corrected this mistake.

• Line 276 mislabels Figure 2B as 2C

28. We have corrected this mistake.
