# Supplementary material for: Immiscible inclusion bodies formed by polyglutamine and poly(glycine-alanine) are enriched with distinct proteomes but converge in proteins that are risk factors for disease and involved in protein degradation
Source: PLoS One. 2020 Aug 28;15(8):e0233247. doi: 10.1371/journal.pone.0233247 (PMC7455042; doi:10.1371/journal.pone.0233247)
Supplement: Attachment — Submitted filename: response to reviewers2.pdf [file pone.0233247.s009.pdf]

Dear Prof van der Wel,

We thank the reviewers for their final suggestions. We have made the suggested changes as indicated below:

Reviewer #2: Most of my comments have been addresses sufficiently. The only point where I am still unhappy is the synergy aspect mentioned in the abstract. Even after the revisions and the additional inclusion of the term protein folding, I fail to see how the results presented here demonstrate a synergetic effect. Perhaps it is possible to phrase this differently.

1. We have removed the word synergy from the abstract and replaced it with “both structures revealed a collection of…”.

Minor points:

Line 19/20: the protein number given in the abstract for proteins associated with both inclusion types is identical to the number given in table S2 for proteins associated with both inclusion types plus the proteins identified for only one kind of inclusion.

2. We thank the reviewer for pointing out this incorrect phrasing and we have now reworded the relevant text in the abstract to more clearly articulate the points.

line 55: I am not sure what mechanism is meant by the phrase “assemble by an amyloid-like mechanism”, and believe that the original phrase that the inclusions were “amyloid-like” better describes what is known about polyQ and polyGA inclusions.

3. We have revised this as suggested.

line 338: please exchange [63,64,65-67] with [63-67]

4. We have revised this as suggested.

lines 585/619: there are links present that are inconsistent with the formatting of the other parts of the reference section

5. We have revised this as suggested.
